# Non-communicable diseases control in China and Japan

**DOI:** 10.1186/s12992-017-0315-8

**Published:** 2017-12-20

**Authors:** Fei Wu, Hiroto Narimatsu, Xiaoqiang Li, Sho Nakamura, Ri Sho, Genming Zhao, Yoshinori Nakata, Wanghong Xu

**Affiliations:** 1 Department of Epidemiology, School of Public Health, Fudan University, 138 Yi Xue Yuan Road, Shanghai, 200032 People’s Republic of China; 2 Key Laboratory of Public Health Safety, Ministry of Education (Fudan University), 138 Yi Xue Yuan Road, Shanghai, 200032 China; 3 Cancer Prevention and Control Division, Kanagawa Cancer Center Research Institute, 2-2-2 Nakao, Asahi-ku, Yokohama, 241-8515 Japan; 4 Department of Clinical Oncology, Faculty of Medicine, Yamagata University, 2-2-2, Iida-nishi, Yamagata, 990-9585 Japan; 5 Department of Public Health, Graduate School of Medical Science, Yamagata University, 2-2-2, Iida-nishi, Yamagata, 990-9585 Japan; 6 Graduate School of Public Health, Teikyo University, 2-11-1 Kaga Itabashi-ku, Tokyo, 1738605 Japan

**Keywords:** Non-communicable diseases, Preventive strategies, Comparison

## Abstract

China and Japan share numerous similarities other than their geographical proximity. Facing the great challenges of non-communicable diseases (NCDs), China and Japan have developed different preventive strategies and systems. While Japan has made great progress in primary prevention of NCDs through strong legislation, the ‘Specific Health Check and Guidance System’ and a unique licensed health professional system, China is attempting to catch up by changing its strategies in NCDs control. In this manuscript, we compared disease burden of NCDs, health care systems and preventive strategies against NCDs between China and Japan. In this light, we summarized the points that the two countries can learn from each other, and proposed recommendations for the two countries in NCDs control.

## Background

China and Japan, other than geographical proximity in eastern Asia, share certain similarities in genetic origins, culture, lifestyles and some other aspects. During past decades, both countries have been facing growing burden of non-communicable diseases (NCDs) due to rapid population ageing, urbanization and lifestyle changes. These similarities indicate that the two countries would have had a similar disease burden of NCDs. However, Japan is a high-income and high-welfare developed country with Gross Domestic Product (GDP) up to $36,285 per capita in 2014 [1]. Its mature health system addressing the growing epidemic of NCDs has achieved a relatively healthy lifestyle and a lower NCD-related premature mortality in general population. China, on the other hand, is a middle-income country with a large population and limited healthcare resources. It is shifting the paradigm from acute to chronic care due to its heavy burden of NCDs and has achieved much in just a few years [2]. Comparison of strategies and efficacy in NCDs control between the two countries may provide valuable global views to improve health care in both countries, particularly for China, and benefit achievement of the Sustainable Development Goals (SDG) of the United Nations [3].

In this review, we compared disease burdens of NCDs, health systems and preventive strategies against NCDs in China and Japan. We searched related literatures online like Pubmed, Web of Science, CNKI and Wanfang using the terms of ‘chronic diseases / non-communicable diseases / NCDs / lifestyle-related diseases’ and ‘prevention / control / health system / strategies’, and obtained NCD-related laws, regulations, guidelines or reports through accessing to the websites of both Chinese and Japanese governments. Based on the comparisons, we summarized the points that the two countries can learn from each other, and proposed recommendations for both countries.

### Disease burden of NCDs in China and Japan

China is a middle-income country experiencing rapid economic growth and urbanization. Increasing life expectancies, tobacco use, harmful use of alcohol, obesity, lack of physical activity, and excess salt, sugar and saturated fats in the diet contribute to the growing incidence and prevalence of NCDs such as heart disease, stroke, diabetes, cancers, and chronic respiratory diseases [4]. In 2012, the prevalence of hypertension and diabetes were 25.2% and 9.7%, respectively, among Chinese adults [5]. The incidence of cancer was 235 per 100,000 in 2013, with lung cancer and breast cancer as the leading causes of death in men and women, respectively [6]. The mortality of NCDs was 533 per 100,000 in 2012, accounting for 87% of all-cause deaths in China [5]. When measured in disability-adjusted life-years (DALY), NCDs accounted for 69% disease burden in 1990 and increased to 77% % in 2010, whereas DALY from communicable, maternal, neonatal, and nutritional disorders declined by 67% during the period [7]. The number of patients with NCDs is projected to double over the coming two decades if absent significant interventions. The disease burden of myocardial infarction, stroke, diabetes, and chronic obstructive pulmonary disease (COPD), the four leading NCDs in China, will rise by 50% during the period of 2010 and 2030 [8]. Nevertheless, as the World Bank estimated, if death rate of cardiovascular diseases (CVD) in China decreases by 1% from 2010 to 2040, the cumulative economic benefits may equal to 68% of actual GDP in 2010, i.e. more than 10.7 trillion dollars [9].

Japan, one of the most developed countries, ranked first in longevity around the world as early as in 1990, and achieved an average life expectancy of 79.9 years for men and 86.3 years for women in 2015 [10]. Japanese have the lowest prevalence of obesity [11, 12] and as low as 19.3% cigarette smoking rate (32.2% in men and 8.2% in women) [13, 14]. Therefore, although NCDs are still the leading causes of death in Japan, mainly stroke (120.6 thousand deaths, or 10.1% NCDs deaths), ischaemic heart diseases (IHD) (102.5 thousand deaths, or 8.6% NCDs deaths) and various cancers, the probability of premature death from CVD, diabetes, COPD and cancers was only 9%. It is reported that deaths caused by CVD and IHD, the top two causes of death in Japan, increased 15.4% and 24.4% from 2005 to 2015, respectively (http://www.healthdata.org/Japan data.org/Japan). Tobacco use and high blood pressure were the two major contributors for adult mortality from NCDs and injuries [15].

Obviously, the situation is severe in both countries. While the prevalence of diabetes and other NCDs in Japan remain stable after entering twenty-first century, those in China are still increasing, which means that China is under much more tremendous pressure. Effective preventive strategies are urgently needed to control NCDs, the main contributor of premature death, particularly in China.

### Preventive strategies of NCDs in Japan and China

#### Legislation and policy

Japan has been administrating law-based governance of NCDs since 1983 when the Health and Medical Services Act for the Aged was enforced to ensure and enhance comprehensive healthcare services for senior citizens. The Act proposed the principle of “Health care at age 40^+^ years, medical care at age 70+ years”, emphasizing the importance of NCDs prevention in middle-aged populations. According to the Act, anyone over 40 years old in Japan would be provided a free healthcare service package which includes: 1) personal health record; 2) health education; 3) health information sharing; 4) health check-up; 5) medical care service; 6) functional training and 7) routine follow-up and guidance by doctors. Following the Act, a series of laws were enacted as supplements. For example, the Long-term Care Insurance Act issued in 1997 stipulates that the elderly over 65 years should be provided with vital organ function evaluation and long-term care services. In 2005, the Community General Support Center was established in each municipality to provide general management of health, welfare and medical care. The Health Promotion Act was implemented in 2004, and the Health and Medical Services Act for the Aged were amended in 2008 as the Act on Assurance of Medical Care for the Elderly. Under these laws, the specific health check-up was initiated to provide service for people aged 40–74 years, greatly advocating health promotion and prioritizing primary prevention of NCDs (Table 1). Based on a national legislation, a unified “Specific Health Check and Guidance System” was concretely implemented by local self-government bodies around the country. Medical care for the elderly over 75 years, on the other hand, was stipulated to be provided by specific long-term care institutions. These laws guaranteed the delivery of health care service and sped up decentralization of the system, making families and communities become the social units responsible for health care for the elderly.

**Table 1:** Framework of *NCDs* control and prevention in Japan

Development	Unhealthy lifestyle →	Risk status →	NCDs →	Rehabilitation
Focuses	Overeating Smoking/Drinking Not exercising Living with stress	Overweight High blood pressure High blood glucose High TC	Cancers Diabetes CVDs Stroke	
Actions	Health Japan 21	Specific health check/guidance Cancer screening	Surveillance Treatment	Self-care Long-term care
Systems	A national health promotion & disease prevention initiative	A mass screening program	Cancer registry Healthcare system • Health insurance • Health care delivery	Long-term care system
Acts/Laws	Health Promotion Act	Act on Assurance of Medical Care for the Elderly Health Promotion Act	Cancer Registration Promotion Act National Health Insurance Act Act on Assurance of Medical Care for the Elderly	The Long-term Care Insurance Act

The legislation on NCDs prevention and control in China lags far behind Japan and other developed countries. Since the New Medical Reform in 2009, China has sped up its pace in legislation in NCDs control. The National People’s Congress, the State Council and the Ministries have formulated a series of laws, regulations and policy documents (Table 2). Up to 2014, a total of 164 national level laws, regulations and policies relevant to NCDs were issued, most of which were after 2009. It is expected that the issues of the laws and regulations may help to decrease risk exposures in Chinese population. However, the legislation in China was affected by many factors, mainly international trend, domestic situation and mainstream concept of decision-makers. For example, China is struggling hard for the Tobacco Control Bill to implement the strategy of Monitor, Protect, Offer, Warn, Enforce and Raise (MPOWER) recommended by the World Health Organization (WHO) [16].

**Table 2:** Major regulatory affairs on *NCDs* control in China

Issuing Institutes	Type of Policies	Topics	Number of Policy Documents			
			Overall	1984-1999	2000-2008	2009 & after
General Office of the Communist Party of China	Decisions, Opinions, Reports, Notices	Medical Reform; Population ageing; Sports; Rural healthcare service; Tobacco control	11	1	5	5
National People’s Congress	Laws and Regulations	Environment protection; Air pollution; Advertisement; Sports; Nationwide fitness program; Food safety; Social insurance; Occupational disease prevention; Minors and the elderly health protection	10	5	1	4
State Council of China	Regulations, Decision, Opinions, Outlines, Reports, Plans, Rules, Notices	Medical Reform; Population ageing and pension service; Health development planning; Planning for national economic and social development; Health service; Food and nutrition; Nationwide fitness program; Patriotic health movement; Mental Health; Medical & healthcare service	29	1	6	22
Ministry of Health	Decree of Minister, Opinions, Guidelines, Standards, Regulations, Protocols, Rules, Outlines, Bulletins, Plans, Reports, Notices	Medical reform; Disease prevention (Cancer, Diabetes, CVD, Dental hygiene, Mental health, Osteoporosis, etc.); Health literacy; Health education; Tobacco control; Health talent team construction; New rural cooperative medical system; Medicines; Medical rescuing; Elderly health; Floating population health	50	0	14	36
Other Minstries	Decree of Minister, Opinions, Notices, Standards, Outlines, Protocols, Rules, Regulations	Medical reform; Medical Insurance; Medical rescuing; Ageing Pension; Student sports; Chinese Medicine; Food safety; Tobacco control; Disabled people health; Community Health; Nationwide fitness program, etc.	64	6	25	33
Total			164	13	51	100

### Priority of NCDs control

Primary prevention is the main strategy for NCDs control in Japan. In 1996, Japan used “lifestyle-related diseases” instead of “adult diseases” to name NCDs, for the diseases are closely related to lifestyles and behaviors. Since then, strategies of NCDs control in Japan changed from secondary prevention focusing on early detection and early treatment or tertiary prevention focusing on patients’ recovery and return to society to primary prevention emphasizing on healthy lifestyle and health promotion. “Healthy Japan 21” further combined detailed, practical and sustainable goals of NCDs control into individuals’ daily life.

The Specific Health Check and Guidance System is the central for NCDs control with a main goal to decrease medical costs. The universal NCDs prevention program for adults aged 40–74 years old was initiated in April 2008 and promoted by the Ministry of Health and the Ministry of Labor and Welfare. According to the health check-up plan, all adults at age 40–74 years old must participate in a routine health check-up which was organized by health insurance societies and implemented in general clinics. The participants are required to provide information on use of medicines, cigarette smoking, any self-conscious symptoms, have body height, weight, waist circumference and blood pressure measured, and have liver function (AST, ALT, γ-GTP), blood lipids and glucose, and several urine markers assayed. The specific health check-up has priority to any other physical examinations, and can be shared by other programs to save medical resources. The results of check-up will be delivered to individuals in form of report sheet by medical insurance societies.

**Fig. 1:**
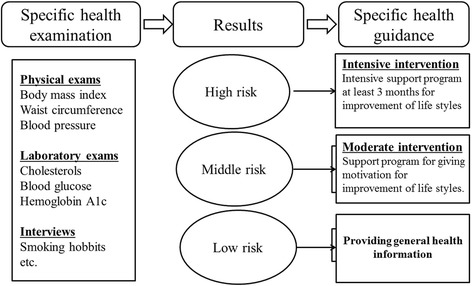
Factors for risk classification in Japan

Based on the results of check-up, all participants were classified into different risk classifications and were provided with different health guidance by medical staff in designated hospitals. The factors for risk classification include cigarette use, waist circumference, blood pressure, blood levels of glucose and cholesterol measured in health check-up [17] (Fig. 1). Qualified staff involving in the program included licensed doctors, hygienists, nutritionists and other professional medical staff. The examinees can choose medical institutions, which introduces a competitive mechanism.

Generally, the measures of NCDs control in China mainly include three aspects, namely, surveys or surveillances of NCDs and related risk factors, health education and health promotion in general population and comprehensive interventions in high-risk population [18]. Besides CVD Surveillance, Cancer Registry and Vital Statistics, a nation-wide sampling survey was conducted every 3 years to estimate prevalence of hypertension, type 2 diabetes, obesity, smoking and other risk factors [19]. For general population, health education is conducted to decrease exposure to tobacco use, unhealthy diet and physical inactivity, and health promotion is implemented to advocate healthy lifestyles. However, due to that health promotion was usually conducted as large-scale, top-down, sharp but short campaigns in China [20, 21], its effect could not be sustainable. For population at high risk, comprehensive interventions (health education, early detection and management of NCDs) are usually implemented by General Practitioners (GP) from grass-roots medical institutes.

Of the three aspects, management of high-risk population is of the most important in China. Taking CVD prevention as an example, the classified management of hypertensive patients is the central task [22]. The classified management of hypertension was first carried out as a routine service in Shanghai, Guangzhou and Beijing, the three biggest cities in China. The basic process is to obtain basic health information such as diagnosis of hypertension, family history, biochemical assay data, physical examination data, and treatment information, then to identify hypertensive patients according to the WHO standard, establish health archives, and classify the patients into I, II and III groups by predicted risk of CVD according to the China Guideline for Hypertension Control. Patients in different groups obtain different frequency and intensity of health education, blood pressure measurement, routine tests of blood and urine, measurements of blood glucose, lipids, liver and kidney functions, electrocardiogram and fundus examinations, behavior interventions, medications and following-up. The risk classification will be adjusted per year [23]. In 2009, China listed the measurement of blood pressure for adults over 35 years at their first visit to any clinic as a national basic public health service, greatly improving awareness of hypertension [24].

The standardized management of NCDs in China is somewhat similar to the “specific health guidance” in Japan. However, the target populations are much different. While Japan focuses on general adult population aged at 40-74 years old, China pays more attention to 260 million prevalent patients with NCDs. Moreover, unlike Japan, China is a country with a large population and huge regional inequity in healthcare resources. It is impossible for China to implement unified policies and strategies of NCDs control with high quality across the whole country. Local governments are encouraged to develop their own strategies and measures on the condition that the basic tasks of NCDs control have been completed.

### Medical insurance

**Fig. 2:**
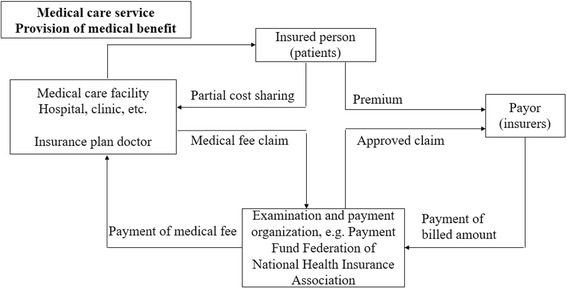
Medical Insurance System in Japan (http://www.mhlw.go.jp/bunya/iryouhoken/iryouhoken01/dl/01_eng.pdf, access to the website on June 19, 2017)

In Japan, the universal health care system covers all citizens according to public health insurance plan which can be broadly divided into two subsystems [17]. The first part is managed by health insurance societies, mutual aid associations and central government, covering employees of large firms, public servants and small or medium firms. The second one is managed by local municipalities and covers self-employed and the elderly [17]. Each hospital or medical clinic in Japan is designated by government as a provider of unified medical care. As shown in Fig. 2, the schedule of fees payable to providers for medical treatment is set by government. Patients access their providers of choice and pay only the co-payment. Then payors reimburse providers for the remainder of the cost via the examination and payment organization [17]. Owing to this system, people can receive universal medical treatment with high quality and low costs, which contributed to the success in health care in Japan in recent 50 years [25].

Social health insurance system in China included urban employee-based basic medical insurance scheme initiated in 1998, the rural new cooperative medical scheme launched in 2003, and urban resident-based basic medical insurance scheme embarked in 2007. The system has developed and expanded very rapidly during past decades and covered almost all citizens in China [26]. While urban employee-based basic medical insurance is mainly supported with payroll taxes, the other two schemes are funded by government subsidies. The system is facing big challenges in addressing NCDs control and treatment. First of all, the system was designed to prioritize inpatient to outpatient service which is the most need for NCDs patients [27]. In most areas of China, the coverage of social health insurance was limited and did not include outpatient costs because the reimbursement is determined mainly by severity of diseases [28]. Moreover, the coverage of insurance schemes, policies of reimbursement and models of supervision and management for NCDs greatly varied across areas in China. In Tianjin and Shenyang, NCDs are considered as special diseases, and the costs for outpatient service can be paid by pooling funds at a certain proportion [29]. Furthermore, Fee for Service (FFS), the most prevalent payment pattern in China, is dependent on quantity rather than quality of healthcare service. Physicians were incented to provide more but dispensable physical examinations, biochemical tests and prescriptions. Therefore, some areas are turning to alternative prospective payment modes like global budget, diagnosis-related groups (DRGs) and capitation [30, 31].

### Multi-sectorial cooperation

Multi-sectorial cooperation in NCDs control in Japan has been well established and practiced. “Health Japan 21” initiated in 2000 was the third national health promotion campaign aimed to establish basic-level professional health care teams. The policies, ideas, and specific goals are included in the "Basic Direction for Comprehensive Implementation of National Health Promotion" which was established by the Minister of Health, Labor, and Welfare in accordance with Article 7 of the Health Promotion Act. Based on the provisions of Article 8 of the Health Promotion Act, each prefecture has taken the country’s fundamental policy into consideration and established its own “Prefectural Health Promotion Plan” as a basic plan for advancing health promotion for its residents. Leadership of central and local governments and active participation of social organizations and individuals guaranteed achievement of the goals (http://www.mhlw.go.jp/seisakunitsuite/bunya/kenkou_iryou/kenkou/kenkounippon21/en/kenkounippon21/).

The practice in Taiyoumura Kashima-gun of Ibaraki Prefecture in Japan provides evidence on the importance of collaborations of governments, industries, societies and individuals in NCDs control. Taiyoumura, Kashima-gun (a part of Hokota city from 2015) in Ibaraki Prefecture, is a village located in northeast of Tokyo, Japan. In the area, the elderly aged over 65 years old accounted for 65% of population. In response to the campaign of the “Health Japan 21” promoted by the central government, the village government and department of health encouraged the villagers, especially the elderly, to participate in an exercise project focusing on muscle activity. Based on a trial including 20 participants of the exercise group averaging 65.2 years old and 23 control subjects averaging 68.4 years, the investigators found that the medical cost was only 148,178 Yen (about 1326 USD) per person per year in intervention group after two-year exercise, much lower than 296,422 Yen (about 2653 USD) per person per year in control group [32].

In China, the tasks of NCDs control were designed to be shared by multiple organizations, including national and local Center for Disease Prevention and Control (CDC), hospitals, Institution of Health Education, and Institute of Maternal and Children Healthcare. In addition, National Cancer Center, National Center of Cardiovascular Disease, Prevention office for Endemic Diseases and other academic organizations are also involved in NCDs control. These organizations provide services on administration, technical guidance and technical consultant in NCDs control, making a vertical networking of classified management with central authorities [33]. All tasks were finally carried out by medical staff in Community Healthcare Service Centers or clinic sites. This top-to-bottom multi-channel management of NCDs control, if not well organized, would cause many problems. Moreover, due to lack of clinical practice, CDCs were weak in providing technical guidance for the Community Healthcare Service Centers; without relevant laws or policies, hospitals and other clinical institutions lack motivation, personnel and budget to take the responsibility in public health and perform effective two-way transfer treatment with communities; for the community healthcare service centers, due to heavy working tasks and lack of incentives, qualified health professionals were quite short in these grass-roots health facilities [34].

China has recognized the importance of multi-sectorial cooperation in NCDs control. In 2012, fifteen Ministries in China jointly issued the "Work plan on NCDs prevention and control in China (2012-2015)" [35] to propose mechanisms of multi-sectorial cooperation and define responsibilities of relevant sectors. The issue of the work plan was a landmark in NCDs control in China. However, due to lack of political incentives and effective mechanisms, coupled with conflicts of interest, multi-sectoral cooperation in NCDs control was just a promise of the central and local governments. Facing the big challenge, the Health and Family Planning Commission of China (Former Ministry of Health) launched the project of “China Healthy Lifestyle for All” initiative in 2007 and the project of “Demonstration Area Construction on NCDs Comprehensive Prevention and Control” in 2011 to explore an appropriate approach to realize multi-sectoral cooperation. The two nation-wide actions have achieved remarkable results in some areas. With the support from local governments, the task of NCDs control has been regarded as a part of local livelihood projects. In these areas, the Departments of Education, Culture, Sports, Propaganda and City administration, as well as various social organizations, worked together to reduce exposure to smoking, unhealthy diet, physical inactivity at population level [36]. By the end of October 2015, a total of 265 national and provincial Demonstration areas distributed in 30 provinces (or Municipalities) have been built [37]. The “Healthy China 2030” issued in 2016 proposed a basic route of “work together and share together”, which requires attempts from both supply-side and demand-side and involvement of societies, industries and individuals to form a powerful force to promote health. The plan will greatly improve multi sectoral cooperation in NCDs control in China.

### Institutional and personnel capacity building

In Japan, all professional healthcare workers are licensed and accredited by the national unified identification system. Professional medical workers including physicians, dentists, pharmacists, plant doctors, public health doctors, dietitians, sports coaches, psychologists and nurses are usually employed by national public health institutes, profit or non-profit organizations. As early as in 2008, there were 286,699 medical doctors, 99,426 dentists, 267,751 pharmacists and 139,700 nurses in Japan, mainly working in hospitals, clinics, healthcare institutes and healthcare centers (http://www.mhlw.go.jp/wp/hakusyo/kousei/10-2/kousei-data/PDF/22010209.pdf). In 2009, a total of 45,603 registered dieticians and 55,326 dieticians in Japan worked in hospitals, schools, canteens, hotels, food industries and government departments (http://www.e-stat.go.jp/SG1/estat/List.do?lid=000001068836). These professionals are responsible for health promotion in the local, and play a central role in spreading health knowledge and directing health behaviors. The public health doctors, dietitians, and sports coaches in local civil services are also involved in health promotion as supplementary. The substantial grass-roots health workers make it easy to implement interventions among Japanese.

China is facing a big challenge in institutional and personnel capacity building. It is reported that China is facing severe shortage of qualified health professionals in grass-roots health facilities [28]. In China, there are over 50,000 community healthcare service centers and more than 100,000 clinic sites in urban and rural areas. However, by the end of 2013, there were only 23,000 GPs with certification in China, far away from the final target of 150,000 during the 12th Five-year Plan for Health [38]. Reconstruction of hierarchical medical system may provide China an opportunity to strengthen its grass root healthcare institutes through capability building.

### Evidence-based policy-making

In Japan, the decision making and implementation of NCDs control are usually based on evidence. According to the Health Promotion Act, Japan has been conducting sampling surveys and established database on population health, behaviors, lifestyle and nutrition. The survey data are usually used to predict epidemic trend of NCDs and related behavior risk factors, and to evaluate effect of prevention and control strategies and measures. Implementation of “Healthy Japan 21” in different areas provided evidence for government to make further decisions. The Japanese government also provides specific funds to support applied researches on NCDs control.

China has established perfect infectious disease reporting system, but not for NCDs. For example, the cancer registry system covers only about 300 cities or counties [6]; the surveillance of behavioral risk factors is carried out every 3 years since 2009 and the cardiovascular diseases registry system is just initiated. Since 1950s, China has only conducted five times national nutrition surveys (in 1959, 1982, 1992, 2004 and 2012, respectively), five times national hypertension surveys (1959, 1979, 1991, 2002, and 2013), four times diabetes surveys (1980, 1984, 1995, and 2008), three times national mortality surveys focusing on cancer death (1973, 1990, and 2006) and four times Tobacco Use Surveys (1984, 1996, 2002, and 2010). Regarding intervention trials, there are only several cancer screening projects through central transfer payments. The openness and sharing of the data from these large-scale surveys and intervention trials is far from enough. Therefore, China lacks long-term, high quality data from basic surveys and large-scale intervention trials to provide evidence for government to make decisions.

### Health information sharing system

Generally, health and medical information sharing is not well-established in Japan. Usually, Japanese have to bring a printed version of their health examination results to visit doctors, which makes it difficult for a physician to obtain sequential results of health examinations. In addition, while electronic medical charts are used in most hospitals, paper medical charts are still used in some hospitals and clinics. Moreover, the electronic medical chart system provided by different system vendors were incompatible, which makes it difficult to establish a health information sharing platform based on available systems. The government is considering to provide each citizen a unique “medical ID” by utilizing the infrastructures of the social security number system (http://www.mhlw.go.jp/stf/seisakunitsuite/bunya/kenkou_iryou/iryouhoken/mynumber/index.html, access to the website on June 19, 2017). In Kanagawa prefecture, personal health record service has been launched. Each individual has his/her personal health record, named “My ME-BYO medical chart”. It includes information of health checks. Such system may solve the problem in sharing information and accelerate utilization of health examination and screening results for clinical and preventive purposes (http://www.pref.kanagawa.jp/cnt/f532715/p991437.html, access to the website on June 19, 2017).

China is attempting to establish an effective and shareable health information system around the country. Zhabei district of Shanghai began to establish health information archives as early as in 2000 as a pilot area. After 10 years’ attempt, a standardized electronic health system was developed to collect and update medical information of individuals from various sources instantly [39]. Based on the regional system, health information can be shared between local communities and secondary hospitals and can be obtained by residents by access to Internet to conduct self-service health management. Due to the success of the health information systems in pilot areas, information platform building was proposed as one of the eight key tasks of the New Medical Reform to improve health care quality and reduce medical costs [40]. The Steering Committee of Health Informatics led by the Ministry of Health published the “Guideline for Building the Regional Health Information Platform based on Electronic Health Records” [34], describing a technical architecture for regional health information platform. Since then, almost all areas in China have initiated electronic health achieves building [41]. Low quality and delayed update of the data, however, have been the main concern and the biggest challenge.

### What China can learn from Japan?

The strategies of NCDs control in Japan and China have their own advantages and disadvantages. China can learn more from Japan for Japan’s mature health system and better effect of NCDs control. However, the different national political and economic conditions determine that China should selectively adopt policies and strategies of Japan, particularly those highly cost-effective from macro perspective, including priority of primary prevention, legislation to control NCDs, cross-sectional cooperation, capacity building for grass-roots institutes, and making healthy lifestyle as civic duty.

### Take primary prevention of NCDs as a priority

Generally, China has been paying much attention to the management of a large number of prevalent patients with NCDs. The grass-root medical staffs were almost occupied by patient registry, health archives establishing, blood pressure measuring and blood sugar testing. They could not find enough time and lack professional ability to conduct effective lifestyle interventions at population level [28]. As a result, the prevalent patients with NCDs increased year by year, and the grass-root medical staff became busier and busier, leading to a vicious circle. It is urgent for China to take primary prevention of NCDs as a priority, just like Japan has done. The rebuilding of hierarchical medical system provides China an opportunity to follow the Japan’s ideal of ‘Health care at age 40^+^ years, medical care at age 70^+^ years’.

### Speed up legislation for NCDs control

To establish a nation-wide healthy social environment is the key for NCDs control in China. To achieve the goal, China should construct legal system to support the establishment of a comprehensive platform, hierarchical medical system, long-term planning, medical insurance system, medical information security, medical information communication and multi-sectorial cooperation in NCDs control. Undoubtedly, Chinese governments will play a central role in the progress.

### Strengthen multi-sectional cooperation

Japan owns a horizontal management system of NCDs under a decentralized model [42]. The system has contributed much to the success of NCDs control in Japan [43]. China should learn from Japan to strengthen horizontal multi-sectional cooperation within current vertical network, making hospitals, CDCs, communities, social organizations and insurance companies work together, implement evidence-based policies, and take primary prevention of NCDs as a priority finally.

### Train grass-root health professionals

China is very short of grass-root medical staff. A large number of health professionals are needed to carry out effective nutrition and exercise interventions and provide psychological counseling in communities, and achieve the goal of primary prevention of NCDs. Therefore, it is urgent for China to develop appropriate human resource policies to attract more qualified health professionals to work at primary health facilities, especially in rural areas. Rather than just to increase salaries, China needs to establish a licensed training system like Japan to increase the number of grass-root medical personnel.

### Make a healthy lifestyle as civic duty

In Japan, according to “Healthy Japan 21”, all objectives in NCDs control can be achieved by detailed, practical and sustainable actions that can be followed in individuals’ daily life. More importantly, Japan has made adopting healthy lifestyles as responsibilities of its citizens. In China, however, there is no any compulsory regulation to urge Chinese citizens to live a healthy life, which has led to epidemic of NCDs and related-risk factors due to low health literacy levels in general population. China should learn from Japan to build a social environment conductive to healthy lifestyle by taking compulsory measures to integrity healthy behaviors in daily life of Chinese citizens.

### Policy implications of China’s experience for Japan

The best part of China experience in NCDs control is its strong self-correct ability and long-term following-up mechanism for NCDs patients. Specifically, policy implications of China experience for Japan include:

### Flexibility in strategy shifting

China has strong error-correcting ability in policy making in NCDs control [33, 44]. Recognizing disadvantages of current health system, China has been adopting pragmatic and experimental approaches within its efficient vertical network of health system, and is trying to rebuild a hierarchical medical system through a process of trial and error, aiming to motivate hospitals to involve in public health and improve primary intervention of NCDs [45].

In Japan, the program of “Specific Health Check and Guidance” has been well designed and carried out for nearly one decade. However, Japan has antiquated and entrenched institutional mechanisms for health policy making [43], which has brought challenges for the country. For example, sputum cytology, a method proved ineffective in decreasing lung cancer mortality, has been using to detect lung cancer in the National Cancer Screening Programme of Japan. It is urgent for Japan to turn to low-dose computed tomography, the only recommended screening test for lung cancer. Furthermore, although the program of“Specific Health Check and Guidance” was suggested to decrease body mass index, blood pressure, blood glucose, blood cholesterols and other clinical outcomes in participants comparing with nonparticipants [17], the results may be greatly overestimated due to serious selection bias. It is reported that only 40%-50% residents participated in the program in recent years [46]. It is necessary to evaluate the program in a scientific designed setting. Moreover, depopulation with rapid ageing is progressing in Japan, particularly in rural areas. Health inequality due to social factors has aroused much concern in the country [14]. Customization of the program of ‘Specific Health Check and Guidance’ is worth investigating and a novel approach is needed to improve efficacy of NCDs prevention in Japan.

### Long-term following-up mechanism for patients with NCDs

In China, an efficient mechanism has been built to actively follow-up patients with cancers, CVD or diabetes [19]. ID number, a unique identification number owned by individuals at birth and remains unchanged in whole life, has been used in diverse health systems in big cities like Shanghai and Beijing, making it easy to passively follow-up patients with NCDs by running record linkage across different electronic health records.

The government of Japan is planning to build a unique “medical ID” based on social security number, which can be used to follow-up NCDs patients and run record linkage (http://www.mhlw.go.jp/stf/seisakunitsuite/bunya/kenkou_iryou/iryouhoken/mynumber/index.html). However, almost 40% hypertensive patients detected in specific health check-up did not visit doctors. More intensive program is needed to urge these patients to visit medical institutions and receive adequate treatment.

### Blueprint for health information sharing system

China has established a health information sharing system in Zhabei district of Shanghai and some other pilot areas [39], and is expanding the system to other areas. Although it is a long way to achieve the goal, China has been aware of importance of the system and sped up the progress. According to the 13^th^ Five-year National Planning on Information, 90% residents in urban and rural areas will have personal electronic health achieves by 2020 (http://www.gov.cn/zhengce/content/2016-12/27/content_5153411.htm).

In Japan, the National Clinical Database has been founded since April 2010 as the parent body of a database system linked to a board certification system [47]. Clinical researches based on the “big data” and derived policy recommendations may have a great impact on businesses, governments and insurance policies. Exchange of respective experience in the area is beneficial for both countries.

## Conclusions

In summary, China and Japan share certain similar strategies in NCDs control such as legislation and policy approaches, standardized management of NCDs and multi-sectorial cooperation in NCDs control, which have been proven effective in other countries and are recommended by the World Health Organization. However, the differences in health system and priorities in NCD control determined the big differences in epidemic status and disease burden of NCDs between China and Japan.

With the rapid economic development, Chinese government is increasing its budget in NCDs control year by year. It is a good opportunity for China to learn from Japan to rebuilt its health system particularly at horizontal level and prioritize primary prevention of NCDs. Facing the big challenge of rapid population ageing and urbanization, Japan also needs to strengthen its health system particularly at vertical level and improve its efficiency in NCDs control. The active exploration and rapid improved situation in NCDs control in China may provide policy implications for Japan. Undoubtedly, both China and Japan can benefit from increasing exchanges in ideals, experiences, knowledge and technologies, and close cooperation in policy making, planning, novel strategies and measures exploring in NCDs control, and finally win the fight against NCDs together.

## Abbreviations

CDC: Center for disease prevention and control
COPD: Chronic obstructive pulmonary disease
CVD: Cardiovascular diseases
DALY: Disability-adjusted life-years
GDP: Gross domestic product
GP: General practitioner
IHD: Ischaemic heart diseases
MPOWER: Monitor, protect, offer, warn, enforce and raise strategy for tobacco control
NCDs: Non-communicable diseases
SDG: Sustainable development goals
WHO: World Health Organization
